# Ultrathin gold nanowires to enhance radiation therapy

**DOI:** 10.1186/s12951-020-00678-3

**Published:** 2020-09-11

**Authors:** Lin Bai, Fangchao Jiang, Renjie Wang, Chaebin Lee, Hui Wang, Weizhong Zhang, Wen Jiang, Dandan Li, Bin Ji, Zibo Li, Shi Gao, Jin Xie, Qingjie Ma

**Affiliations:** 1 Department of Nuclear Medicine, China-Japan Union Hospital of Jilin University, Changchun, 130033 Jilin China; 2 NHC Key Laboratory of Radiobiology, School of Public Health of Jilin University, Changchun, 130033 Jilin China; 3 Department of Chemistry, University of Georgia, Athens, GA 30602 USA; 4 Department of Radiology, University of North Carolina at Chapel Hill, Chapel Hill, NC 27599 USA; 5 Department of Gastrointestinal Medicine, Endoscopy Center, China-Japan Union Hospital of Jilin University, Changchun, 130033 Jilin China

**Keywords:** Gold nanoparticles, Radiation therapy, Radiosensitizer, Radicals, Nanowires

## Abstract

**Background:**

Radiation therapy is a main treatment option for cancer. Due to normal tissue toxicity, radiosensitizers are commonly used to enhance RT. In particular, heavy metal or high-Z materials, such as gold nanoparticles, have been investigated as radiosensitizers. So far, however, the related studies have been focused on spherical gold nanoparticles. In this study, we assessed the potential of ultra-thin gold nanowires as a radiosensitizer, which is the first time.

**Methods:**

Gold nanowires were synthesized by the reduction of HAuCl_4_ in hexane. The as-synthesized gold nanowires were then coated with a layer of PEGylated phospholipid to be rendered soluble in water. Spherical gold nanoparticles coated with the same phospholipid were also synthesized as a comparison. Gold nanowires and gold nanospheres were first tested in solutions for their ability to enhance radical production under irradiation. They were then incubated with 4T1 cells to assess whether they could elevate cell oxidative stress under irradiation. Lastly, gold nanowires and gold nanoparticles were intratumorally injected into a 4T1 xenograft model, followed by irradiation applied to tumors (3 Gy/per day for three days). Tumor growth was monitored and compared.

**Results:**

Our studies showed that gold nanowires are superior to gold nanospheres in enhancing radical production under X-ray radiation. In vitro analysis found that the presence of gold nanowires caused elevated lipid peroxidation and intracellular oxidative stress under radiation. When tested in vivo, gold nanowires plus irradiation led to better tumor suppression than gold nanospheres plus radiation. Moreover, gold nanowires were found to be gradually reduced to shorter nanowires by glutathione, which may benefit fractionated radiation.

**Conclusion:**

Our studies suggest that gold nanowires are a promising type of radiosensitizer that can be safely injected into tumors to enhance radiotherapy. While the current study was conducted in a breast cancer model, the approach can be extended to the treatment of other cancer types.
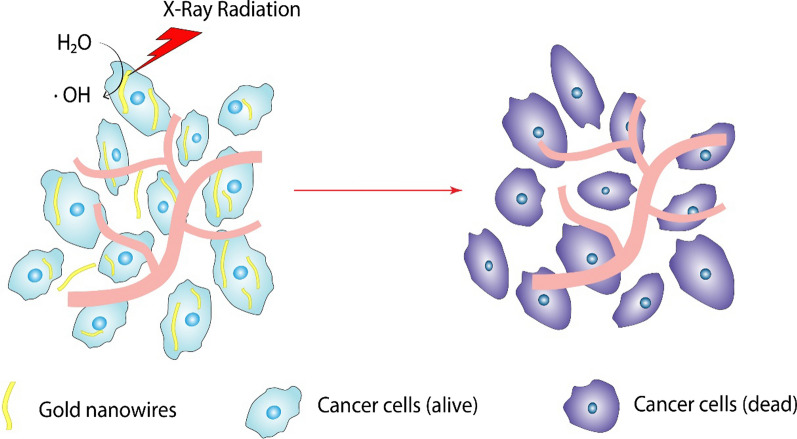

## Background

External radiotherapy (RT) remains a mainstay cancer treatment option. RT exploits ionizing radiation such as X-rays to damage DNA and lipids, eventually leading to cancer cell death [1, 2]. However, the amount of radiation one can receive is limited by normal tissue toxicity [3]. To improve tumor control at a given radiation dose, radiation modifiers or radiosensitizers are often used during RT [4]. One promising type of radiosensitizer is heavy-metal or high-Z nanoparticles [5]. Possessing heavy elements and large mass energy coefficients, high-Z nanoparticles can enhance photoelectric interactions and Auger effects, increasing energy deposition in tumors and improving RT outcomes [5–8]. To maximize radiosensitizing effects, it is currently a common practice to directly administer high-Z nanoparticles into tumors. One example is intratumoral hafnium oxide nanoparticles, which are being tested in the clinic for enhancing RT against cancer types such as soft tissue sarcoma and head and neck cancer [9–11]. Another widely studied high-Z element is gold. A number of groups have observed radiosensitizing effects with gold nanoparticles under both kV and MV beams [12–14]. So far, most of these studies are performed with gold nanospheres (GNSs) [12–14], with gold nanorods [15–17] and nanospikes [16] occasionally tested.

**Scheme 1.**
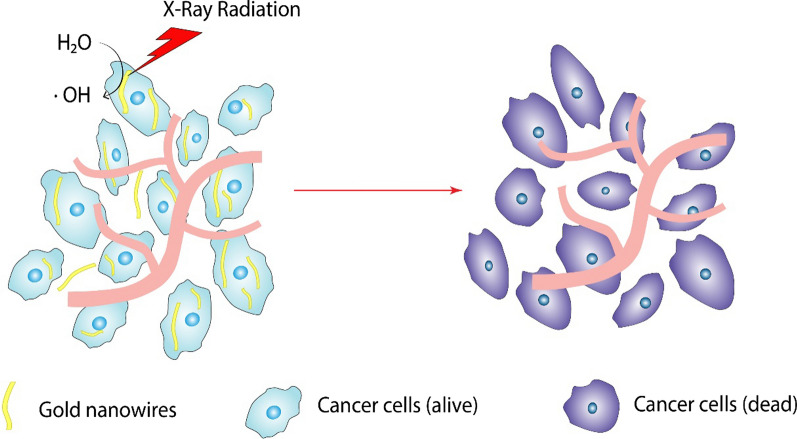
Schematic illustration of GNWs-based radiosensitizing effects that enhance RT

Herein we investigate gold nanowires (GNWs) as a novel type of high-Z radiosensitizer (Scheme 1). GNWs are a one-dimensional nanomaterial with a narrow diameter (< 5 nm) and a very long length (e.g. hundreds nm to several microns; as a comparison, nanorods are typically shorter than 20 nm). Previously, the Sun [18] and Yang [19] groups reported the synthesis of GNWs. To the best of our knowledge, however, there have been few or none attempts of exploring the bio-applications of GNWs. One challenge has been that newly-synthesized GNWs are hydrophobic on the surface and cannot be dispersed in aqueous solutions. Herein we solved this issue by coating GNWs with a PEGylated phospholipid, 1,2-distearoyl-sn-glycero-3-phosphoethanol-amine-*N*-[amino(polyethylene glycol)-2000], or DSPE-PEG (2000) Amine. We tested the resulting GNWs in solutions and in vitro in 4T1 cells, with the postulation that GNWs may improve radical production in aqueous solutions thereby enhancing cell killing under irradiation. For comparison, DSPE-PEG (2000) Amine coated GNSs were also prepared and tested. Lastly, we intratumorally injected GNWs and GNSs into 4T1 tumor bearing mice, and investigated their radiosensitizing effects in vivo.

## Results

**Fig. 1:**
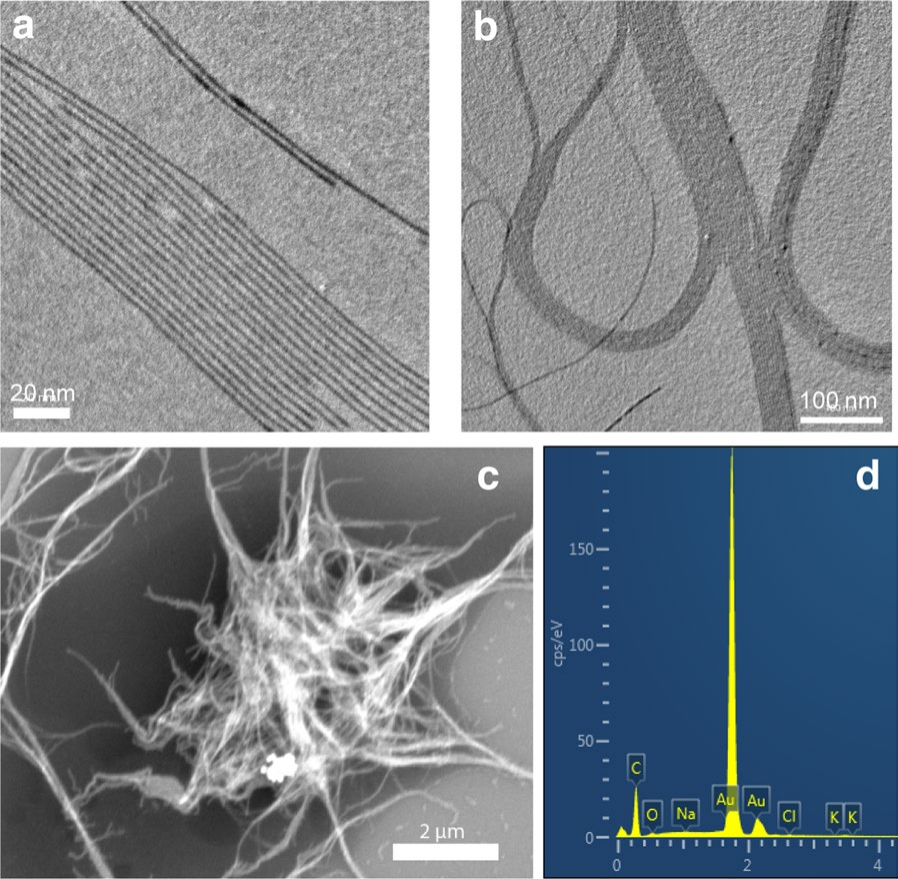
**a**, **b** TEM image of GNWs. Long GNWs tend to form bundles when deposited onto a substrate. **c** SEM image of GNWs. Scale bar, 2 μm. **d** EDS spectrum of GNWs. Au is the main element in the nanostructure

**Fig. 2:**
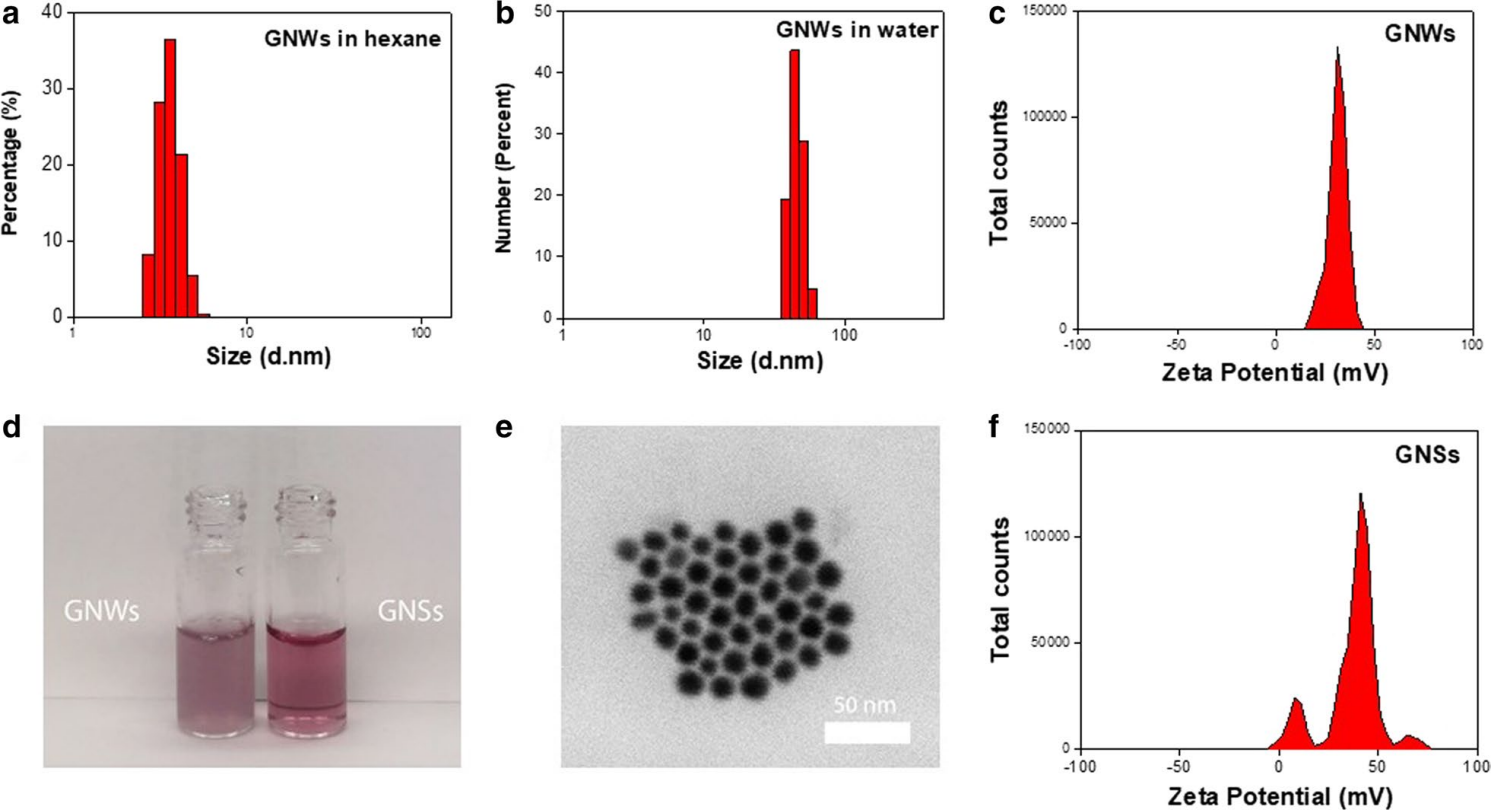
**a** DLS of GNWs in hexane. **b** DLS of phospholipid-coated GNWs in water. **c** Zeta potential of phospholipid-coated GNWs in water. **d** Photos of GNWs and GNSs dispersed in water. **e** TEM image of GNSs. Scale bar, 50 nm. **f** Zeta potential of phospholipid-coated GNSs in water

We synthesized GNWs following a published protocol with modifications [19]. Transmission electron microscopy (TEM) found that the as-synthesized GNWs had a diameter of ~ 3.6 nm and a length of 1–3 μm (Fig. 1a, b, Additional file 1: Figure S1). GNWs tend to form bundles when deposited onto a substrate and the solvent evaporated. This was visualized by both TEM and scanning transmission electron microscopy (STEM) (Fig. 1b, c). Energy Dispersive Spectroscopy (EDS) elemental analysis confirmed that the nanowires were made of gold (Fig. 1d). Dynamic light scattering (DLS) showed that the hydrodynamic size of GNWs in hexane was ~ 4 nm (Fig. 2a), which agrees well with the individual diameter of the nanowires (Fig. 1a, b).

As-synthesized GNWs were coated with a layer of oleylamine and their surface was hydrophobic. These GNWs can be dispersed in non-polar solvents like hexane and chloroform but not in water. To render these GNWs dispersible in aqueous solutions, we coated GNWs with PEGylated phospholipid. The amphiphilic DSPE-PEG (2000) Amine was imparted onto the surface of GNWs through hydrophobic–hydrophobic interaction, with the PEG chains sticking outward to interact with water molecules, which supports particle suspension. The resulting, DSPE-PEG (2000) Amine coated GNWs were readily dispersed in aqueous solutions such as a phosphate-buffered saline (PBS) (Fig. 2b). DSPE-PEG (2000) Amine was chosen over other coating materials because the surface modification procedure is straightforward and mild, which is highly important because GNWs are extremely fragile [18, 19]. Moreover, the primary amine head group would render the nanoparticle surface positively charged, which favors fast and efficient cell membrane attachment. DLS showed that the phospholipid coated GNWs were 45 nm in PBS (Fig. 2b), with a positive surface charge of + 31.1 mV (Fig. 2c). The resulting solution resembles that of GNSs but has a purple hue (Fig. 2d). TEM found that GNWs remained well dispersed after surface modification but the length was shortened (Additional file 1: Figures S1, S2), which again underscores the fragility of GNWs. For comparison, we also synthesized GNSs [20], and coated them with DSPE-PEG(2000) Amine (Fig. 2d). The resulting GNSs have an average diameter of 14 nm (Fig. 2e) and a similar positive surface charge of + 37.1 mV (Fig. 2f).

**Fig. 3:**
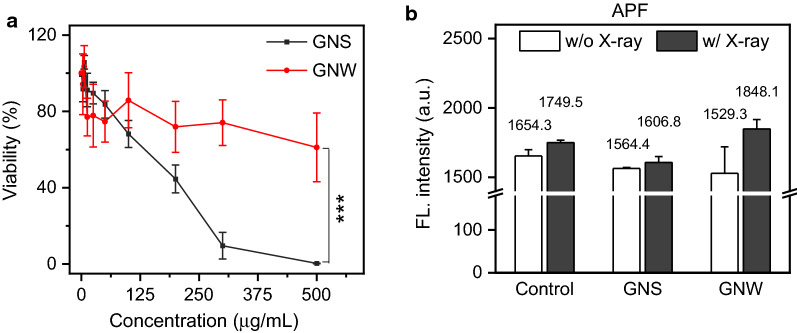
**a** Cytotoxicity of GNWs and GNSs in the absence of radiation, measured by ATP assay at 24 h. 4T1 cells were incubated with GNWs and GNSs at different concentrations (3.125–500 μg Au/mL) for 24 h. PBS treated cells were studied as a control. ***p < 0.001. **b** Hydroxyl radical generation under RT in the presence of GNWs or GNSs (50 μg/mL), measured by APF assay

We then analyzed the cytotoxicity of GNWs and GNSs using ATPlite Viability Assay in the absence of radiation. We incubated GNWs or GNSs with 4T1 cells at different concentrations (3.1–500.0 μg/mL, gold concentration, the same below). After 24 h incubation, the cells were washed, incubated with an ATPlite solution for 10 min, and the luminescent signals were analyzed on a microplate reader. For both GNWs and GNSs, the toxicity was low when the concentration was below 50 μg/mL (Fig. 3a), where the cell viability remained above 80%. Interestingly, GNSs induced a significant cell viability drop when their concentration was above 100 µg/mL, while GNWs showed greater biocompatibility (Fig. 3a). Such toxicity of positively-charged GNSs at high concentrations was also observed by others [21–23].

We next examined the radiosensitizing effects of GNWs. We first tested radical generation in PBS solutions of GNWs or GNSs (50 μg/mL), with and without 5 Gy irradiation (the same dose was used for all solution and in vitro studies). This was quantified with aminophenyl fluorescein (APF), a florigenic sensor that is specific to hydroxyl radicals [24]. While ionizing irradiation can generate a broad range of radicals, hydroxyl radical is the most relevant in RT as water is the most abundant molecule in cells/tissues [25, 26]. APF assay showed that the presence of GNWs increased hydroxyl radical production by 20.8% (Fig. 3b), relative to that of 2.7% for GNSs (Fig. 3b). This is likely attributed to the relative large surface area of the thin nanowires (see Additional file 1 for detailed calculations) and their intrinsic anisotropic morphology with high surface atoms [27–29], which may promote radical production.

**Fig. 4:**
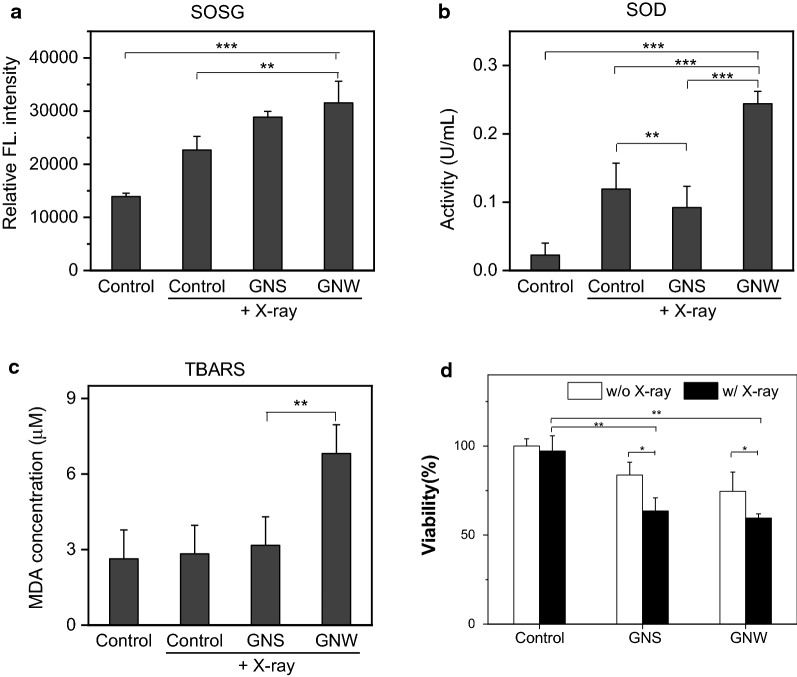
**a** SOSG, **b** SOD, and **c** TBARS assay with 4T1 cells after they were incubated with GNWs or GNSs (50 μg/mL) and then irradiated (5 Gy). **d** Cytotoxicity of GNWs + RT and GNSs + RT (50 μg/mL, 5 Gy), assessed by ATP assay with 4T1 cells. *p < 0.05; **p < 0.01; ***p < 0.001

We then assessed GNWs + RT in vitro, with a focus on their impact on intracellular radical level changes. This was assessed with Singlet Oxygen Sensor Green (SOSG) assays in 4T1 cells [30–34], a murine breast cancer cell line. Our results showed that X-ray alone increased ^1^O_2_ level by 62.9% relative to the control (Fig. 4a). In the presence of GNSs and GNWs, the ^1^O_2_ level was further elevated, displaying an increase of 107.4% and 126.6%, respectively, relative to the control cells. This enhancement was attributed to gold-promoted radical production and in line with the observations made with solutions. We also assessed the impact of this enhanced radical production on cell oxidative stress (Fig. 4b). GNWs plus radiation drastically elevated cellular superoxide dismutase (SOD) activity to 0.244 U/mL, compared to 0.119 U/mL for RT only. As a comparison, GNSs plus X-ray did not significantly increase the SOD level compared to the radiation only control (0.092 U/mL). The increase in oxidative stress was also confirmed by thiobarbituric acid reactive substances assay or TBARS assay, which measures the level of lipid oxidation [35, 36] (Fig. 4c). Specifically, untreated 4T1 cells showed a malondialdehyde (MDA) level of 2.63 µM. This number barely changed when cells were incubated with GNWs or GNSs (2.83 and 2.65 µM, respectively). GNSs + RT led to a small increase of MDA level relative to RT alone (3.17 and 2.83 µM, respectively). Much greater lipid oxidation was observed when GNWs were used in combination with RT, in which case the MDA level was elevated to 6.81 μM.

Subsequently, we assessed whether GNWs facilitate RT-induced toxicity. This was evaluated in 4T1 cells using ATPlite assays (Fig. 4d). Briefly, we incubated 4T1 cells with either GNWs or GNSs at 50 µg/mL for 24 h. According to cytotoxicity studies performed in the absence of irradiation (Fig. 3a), neither GNWs nor GNSs should not cause severe toxicity at this concentration. We then applied 5 Gy radiation to the cells, and conducted ATPlite assay 24 h later. We found enhanced toxicity for both nanomaterials (Fig. 4d). Specifically, while RT alone had minimal impact of cell viability at 24 h, GNWs + RT and GNSs + RT reduced the viability to 59.6% and 63.5%, respectively, relative to the control.

**Fig. 5:**
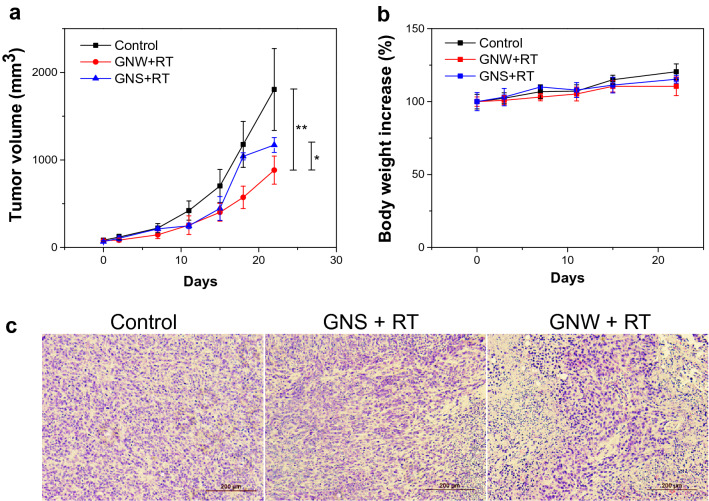
In vivo RT studies with GNWs or GNSs. **a** Tumor growth curves. 4T1 bearing mice were i.t. injected with GNWs or GNSs (1 mg/mL in 70 µL PBS). PBS alone was studied as a control. *p < 0.05; **p < 0.01. **b** Tumor growth curves. **c** H&E staining of tumor tissues taken from treated animals. Scale bars, 200 µm

Lastly, we examined GNWs + RT in vivo in 4T1 subcutaneous tumor models. Briefly, we inoculated 2 × 10^5^ 4T1 cells to the right flank of BALB/c mice. When tumor size reached ~ 100 mm^3^, we intratumorally (i.t.) injected GNWs or GNSs (1 mg Au/mL in 70 µL PBS) into the mice (n = 5). We then delivered three doses of irradiation (3 Gy × 3, single beam radiation delivered through an X-RAD 320 system) to tumors on Day 0, 1 and 2, with the rest of the animal body lead-shielded. For controls, PBS at the same volume was i.t. injected. GNWs + RT efficiently slowed down tumor growth, leading to a tumor inhibition rate (TIR) of 212.5% on Day 22 (Fig. 5a). This was superior to GNSs + RT, which showed a TIR value of 35.7% (*p* < 0.05, Fig. 5a). We euthanized the animals on Day 22, and harvested tumors for histological analysis (Fig. 5c). The H&E staining results overall agree with the tumor growth trend, with the GNWs + RT group showing a significantly reduced cancer cell population. Meanwhile, in both treatment groups, we observed no sign of acute toxicity. No weight loss was observed throughout the whole experiment (Fig. 5b).

## Discussions

In this study, we investigated the potential of GNWs as a novel type of radiosensitizer, which is the first time. Compared with GNSs, we observed an increased production of radicals under radiation in solutions and in cells. Although GNWs may be too long for direct cellular uptake, GNWs are superior to GNS at enhancing cellular oxidative stress under radiation. This is probably attributed to cell-surface-bound GNWs that facilitate lipid damage under radiation, a hypothesis that was supported by the TBARS assay results.

In a separate study, we tested the stability of GNWs in a medium containing glutathione (1 mM), a reducing agent that is abundant in the extracellular milieu. Our studies showed that 530-nm absorbance, the characteristic peak for the transverse surface plasmon resonance, was increased over time (Additional file 1: Figure S3a). This indicates gradual degradation of GNWs that results in the formation of shorter nanowires or even GNSs. This hypothesis was supported by TEM analysis, finding GNWs of reduced lengths and an increased number of GNSs over incubation (Additional file 1: Figure S3b). Unlike small GNSs that may be quickly cleared from tumors after injection, relatively bulky GNWs may retain in tumors and serve as a reservoir for sustained release of gold nanostructures of smaller dimensions. This a unique feature could be another advantage of GNWs, given that longitudinal, fractionated irradiation is often required for clinical RT.

It is possible to combine GNWs with small molecule therapeutics to further enhance RT efficacy. For instance, the di-layered lipid coating on GNWs can be potentially loaded with chemotherapeutics such as 5-FU and paclitaxel. The resulting GNWs may gradually release small molecule agents that may synergize with the high-Z radiosensitizing effects of gold for maximum tumor control. Meanwhile, there have been extensive reports on exploiting gold nanoparticles as a medium to convert radio frequency (RF) [37, 38] or visible/near-infrared [39, 40] photon energy to heat. It will be interesting to explore if GNWs can function as a nanotransducer to mediate RF- or photo-based thermal therapy in addition to sensitizing cancer cells to RT.

## Conclusion

Overall, our studies suggest the great potential of GNWs as a novel type of radiosensitizer. Compared to GNSs, GNWs showed lower toxicity in the absence of radiation but higher efficiency at enhancing RT. While the current study is performed in breast cancer models, the approach can be potentially extended to the treatment of other cancer types such as brain, prostate, and head and neck cancer.

## Methods

### Materials

Gold chloride trihydrate (HAuCl_4_·3H_2_O, ≥ 99.9%, Sigma), tri-isopropylsilane (C_9_H_22_Si, 98%, Sigma), l-Glutathione reduced (C_10_H_17_N_3_O_6_S, ≥ 98.0%, Sigma), hexane (C_6_H_14_, ≥ 99%, Sigma), oleylamine (C_18_H_35_NH_2_, technical grade, 70%, Sigma), toluene (C_7_H_8_, anhydrous, 99.8%, Sigma), Methylene blue (C_16_H_18_ClN_3_S·xH_2_O, powder, ≥ 82%, Sigma) were purchased from Sigma-Aldrich. The other chemicals include chloroform (CHCl_3_, ≥ 99.8%, Fisher Scientific), 3′-(*p*-aminophenyl) fluorescein (APF, Life Technologies), Singlet Oxygen Sensor Green (SOSG, Life Technologies), Superoxide dismutase assay kit (SOD, Cayman Chemical), Phosphate Buffer saline (PBS, pH 7.2), Milli-Q Water (H_2_O, 18.2 MΩ cm@25 ℃), ATPlite 1step kit (ATP, PerkinElmer), TBARs assay kit (TBARs, Cayman Chemical).

### Synthesis of gold nanowires (GNWs)

The gold nanowires were synthesized according to a published protocol with modifications [19]. Briefly, 22 mg HAuCl_4_∙3H_2_O was mixed with 0.738 mL of oleylamine and 20 mL of hexane. The solution was vigorously stirred at room temperature until a homogeneous solution was formed. 1.03 mL of triisopropylsilane was then added into the solution and mild stirring was applied. The final solution was kept still at room temperature for 12 h. Gold nanowires were collected by centrifugation at 6000 rpm for 20 min and repeatedly wash with a toluene/ethanol 1:1 volume ratio mixture. The final product can be store in 10 mL of toluene or hexane.

### Synthesis of gold nanospheres (GNSs)

Gold nanospheres were synthesized according to a published protocol [20]. Typically, 0.2318 g HAuCl_4_∙3H_2_O was mixed with 2 mL hexane and 10 mL oleylamine in a 50 mL flask. The solution was kept at 80 °C under vigorous stirring and N_2_ gas was applied to evaporate extra hexane. After 5 min, the stirring was stopped and the solution kept still at 80 °C for 5 h. Nanospheres can be collected by adding 10 mL ethanol and centrifuge at 7000 rpm for 5 min. The final product was washed three times with ethanol, and finally stored in 10 mL hexane.

### Lipid coating of gold nanoparticles

The as-synthesized GNWs or GNSs were coated with a layer of PEGylated phospholipid. Typically, 200 µL of GNWs or GNSs solution was diluted with 5 mL hexane. 160 µL of DSPE-PEG(2000) Amine in chloroform (1 mg/mL) was then added into the solution. The solution was stirred at room temperature for half an hour. Then the solvent was removed by rotorvap. Milli-Q Water or PBS was finally added to the vessel to redisperse the lipid coated gold nanoparticles.

### Characterizations of gold nanoparticles

The hydrodynamic size and surface charge of the particles were characterized by DLS and Z-potential. The morphology and EDS element mapping of nanoparticles were assessed by both Scanning Electron Microscope (SEM, FEI Teneo) equipped with a EDAX EDS system and Transmission Electron Microscope (TEM, FEI Tecnai20, 200 kV).

### Cell culture

4T1 breast cancer cells were used for in vitro and in vivo studies. Cells were grown in RPMI1640 medium supplemented with 10% FBS and 100 units/mL of penicillin (ATCC). The cells were maintained in a humidified, 5% carbon dioxide (CO_2_) atmosphere at 37 °C.

### Cell viability

Cell viability was evaluated by ATP assay with 4T1 cells. Briefly, 4T1 cells with an initial density of 5000 cells/well were seeded in a 96-well plate. After 24 h’s inoculation, the incubation medium was aspirated and replaced with RPMI solutions containing different concentrations of GNWs or GNSs. After another 24 h’s incubation, incubation medium was aspirated. 50 µL ATPlite 1 step substrate solution was mixed with 50 µL RPMI medium and the mixture was added into each well. The plate was sealed and mixed for 10 min at room temperature before test. Luminescence signals were measured using a microreader (Biotek). Average luminescence intensity was computed and compared.

For treatment with nanoparticles + X-ray, 4T1 cells with an initial density of 5000 cells/well were incubated in 96-well plates. 24 h after the inoculation, cell medium was replaced with 100 µL RPMI medium solutions containing GNWs or GNSs. 5 Gy X-ray radiation was applied 24 h after the incubation. 24 h after the irradiation, cell medium was replaced with a mixture of 50 µL of ATPlite 1 step substrate solution and 50 µL RPMI. The plate was sealed and mixed for 10 min at room temperature before test. Luminescent signals were measured on a microreader (Biotek). Average luminescence intensity was computed and compared.

### Singlet oxygen quantification by SOSG

SOSG assay was performed by following vendor’s protocol (ThermoFisher). Typically, 100 µg SOSG was dissolved in 33 µL methanol to make a 5 mM stock solution. The solution was diluted with Milli-Q water to 5 µM test solution before use. 4T1 cells were incubated in 96-well plates with an initial density of 5000 cells/well. 50 µg/mL in 100 µL incubation medium was added after 24 h’s inoculation. After another 24 h’s of incubation, 5 Gy X-ray radiation was applied. Immediately after irradiation, cell medium was aspirated and replaced with 100 µL RPMI containing 5 µM SOSG. The plate was kept in the dark at room temperature. Fluorescence signals were measured on a microreader (Biotek). Excitation/emission wavelength were set at 504/525 nm. Similar protocol was used for SOSG studies with nanoparticle solutions.

### SOD assay

SOD activity was assessed by following the vendor’s protocol (Cayman Chemical). Assay buffer, sample buffer, radical detector, SOD standard, and Xanthine oxidase were from the vendor. 4T1 cells were incubated in 96-well plates with an initial density of 5000 cells/well. 100 µL medium containing 50 µg Au/mL GNWs or GNSs were incubated with the cells for 24 h. 5 Gy X-ray radiation was applied. Immediately after irradiation, medium was aspired and cells rinsed with sample buffer. 200 µL diluted Radical Detector was mixed with 10 µL sample buffer solution and added to each well. Reaction was initiated with 20 µL Xanithine Oxidase solution and the 96-well plate was kept in dark at room temperature and shaken for 10 min before test. For standards, the provided standard solution was diluted with sample buffer and 10 µL diluted sample buffer solution was mixed with 200 µL diluted Radical Detector and 20 µL diluted Xanthine Oxidase. Signals were measured on a microreader (Biotek). Absorbance at 450 nm was measured and compared to a standard curve.

### Hydroxyl radicals in solutions

Hydroxyl radical generation was characterized by APF assay according to a vendor’s protocol (ThermoFisher). A 20 µL 5 mM stock APF solution was diluted with PBS to make a 2 µM testing solution before test. 100 µL testing solution containing GNWs or GNSs were exposed to 5 Gy X-ray radiation and kept in the dark for 30 min. The resulting solution was finally diluted with same amount of fresh PBS and the fluorescence (490/515 nm) was measured on a microreader (Biotek).

### Lipid peroxidation

TBARs assay kit was used to detect malondialdehyde (MDA), a product of lipid peroxidation. 4T1 cells were incubated in 96-well plates with an initial density of 5000 cells/well for 24 h. After that 100 µL medium containing 50 µg Au/mL GNWs or GNSs were incubated with the cells for another 24 h. After incubation with nanoparticles cells were irradiated with X-ray with dose of 5 Gy. Immediately after irradiation, the medium was aspirated and cells were rinsed with PBS buffer. Cells were homogenized on ice and the protein was collected and determined by BCA method. In each sample, equal amount protein was mixed with TBARs reagents (50 µL sodium dodecyl sulfate (SDS) solution and 2 mL color reagent) and samples vials were then placed in boiling water for 1 h. After the reaction, the vails were placed immediately in ice bath for 10 min to stop the reaction. After 10 min, the vials were centrifuged for 15 min at 1600*g*, 4 °C. 150 µL of the solution was then transferred into a new 96-well plate and data were collected by reading the absorbance at 530 nm and emission at 550 nm with the help of a microplate reader (Biotek).

### In vivo study

Animal studied were performed according to a protocol approved by the Institutional Animal Care and Use Committee (IACUC) of the University of Georgia. The animals were maintained under pathogen-free conditions. 4 T-1 tumor model was generated by subcutaneously injecting 2 × 10^5^ cells in 50 μL PBS into the right flank of 5–6 week old female BALB/c mice (Charles River). All the mice were randomly divided into 3 groups (n = 5). When the average tumor volume was about 100 mm^3^, 70 μL GNWs or GNSs in PBS (1.0 mg/mL) were intratumorally injected into the mice on Day 0. The injection was performed at five sites of the tumor to ensure good coverage. Three doses of irradiation (3 Gy × 3) through an X-RAD 320 system to tumors on Day 0 (1 h after injection), 1 and 2. PBS treated group with or without radiation was used as control. The tumor size and body weight were inspected every 3 days. The tumor was measured in two dimensions with a caliper, and the tumor volume was estimated as (length) × (width)^2^/2. After 28 days, animals were euthanized and the tumor were dissected into slices for H&E staining.

### Statistical analysis

For in vitro study, all measurements were performed at least three times. Measured values were presented as mean ± SD. One tailed Student's *t* test was used for comparison among groups, with *p* values of 0.05 or less representing statistical significance.

## Supplementary information

**Additional file 1.** Additional Figures, Figure S1–S3.

## Data Availability

Additional file is available online or by request.
