# Correction to: Ultrathin gold nanowires to enhance radiation therapy

**DOI:** 10.1186/s12951-021-00953-x

**Published:** 2021-07-19

**Authors:** Lin Bai, Fangchao Jiang, Renjie Wang, Chaebin Lee, Hui Wang, Weizhong Zhang, Wen Jiang, Dandan Li, Bin Ji, Zibo Li, Shi Gao, Jin Xie, Qingjie Ma

**Affiliations:** 1 Department of Nuclear Medicine, China-Japan Union Hospital of Jilin University, Changchun, 130033 Jilin China; 2 NHC Key Laboratory of Radiobiology, School of Public Health of Jilin University, Changchun, 130033 Jilin China; 3 Department of Chemistry, University of Georgia, Athens, GA 30602 USA; 4 Department of Radiology, University of North Carolina at Chapel Hill, Chapel Hill, NC 27599 USA; 5 Department of Gastrointestinal Medicine, Endoscopy Center, China-Japan Union Hospital of Jilin University, Changchun, 130033 Jilin China

## Correction to: J Nanobiotechnol (2020) 18:131 https://doi.org/10.1186/s12951-020-00678-3

Following publication of the original article [1], the authors identified an error in Fig. 5c.

The correct Fig. 5c and its caption is given in this erratum.

**Fig. 5:**
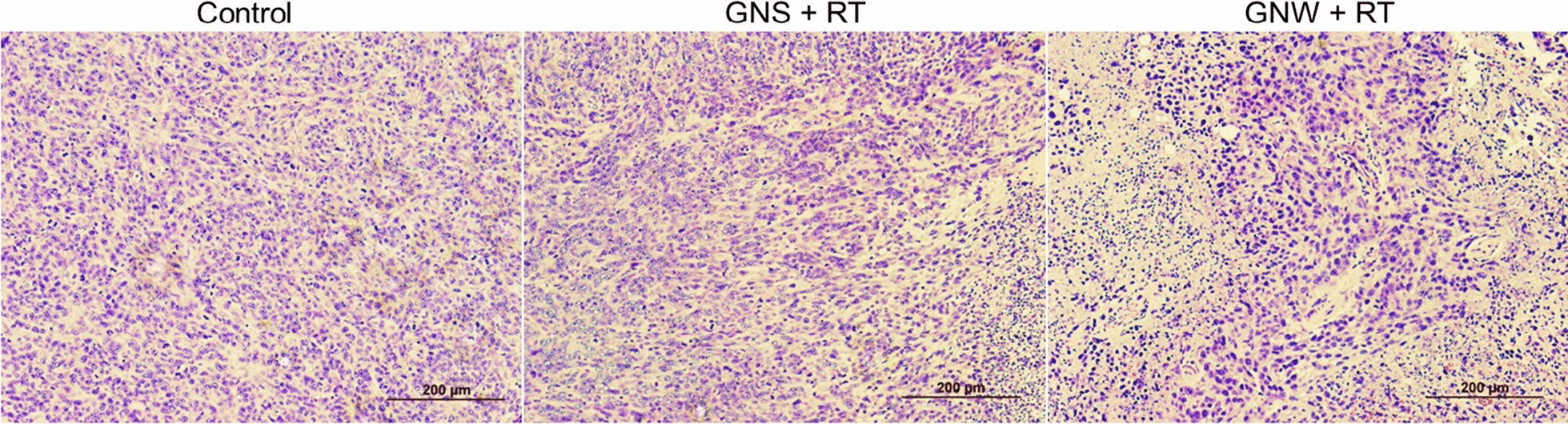
**c** H&E staining of tumor tissues taken from treated animals. Scale bars, 200 µm

The original article has been revised.
