# Supplementary material for: Ultrathin gold nanowires to enhance radiation therapy
Source: J Nanobiotechnology. 2020 Sep 11;18:131. doi: 10.1186/s12951-020-00678-3 (PMC7488570; doi:10.1186/s12951-020-00678-3)
Supplement: Supplementary file 1 — Additional file 1. Additional Figures, Figure S1–S3. [file 12951_2020_678_MOESM1_ESM.pdf]

**Supporting Information for**

# Ultrathin Gold Nanowires to Enhance Radiation Therapy

*Lin Bai[1,2,†], Fangchao Jiang[3,†], Renjie Wang[1,2,†], Chaebin Lee[3], Hui Wang[4], Weizhong Zhang[3], Wen Jiang[3], Dandan Li[5], Bin Ji,[1,2] Zibo Li[4], Shi Gao[1,2], Jin Xie[3,*], Qingjie Ma[1,2*]*

[1] Department of Nuclear Medicine, China–Japan Union Hospital of Jilin University, Changchun, Jilin, 130033, China

[2] NHC Key Laboratory of Radiobiology, School of Public Health of Jilin University, Changchun, Jilin, 130033, China

[3] Department of Chemistry, University of Georgia, Athens, Georgia 30602, USA

[4] Department of Radiology, University of North Carolina at Chapel Hill, Chapel Hill, NC 27599, USA

[5] Department of Gastrointestinal Medicine, Endoscopy Center, China-Japan Union Hospital of Jilin University, Changchun, Jilin, 130033, China

* Corresponding authors: jinxie@uga.edu (J. X.), maqj@jlu.edu.cm (Q. M.)

† These authors contributed equally to this work.

**Surface area calculation:** To compare the surface area of GNWs and GNSs, we assume GNSs are spherical with a radius of 7 nm and GNWs are 3.6 nm in diameter and 100-nm in length. We assume that Au atoms in both structures are tightly complied and that the volume of nanoparticles is proportional to the mass of Au.

The surface area of GNS will then be:

$S_{GNSs}= 4\pi r^2 = 4\times 49\pi = 615.752$ nm$^2$

The surface area of GNWs will be:

$S_{GNWs}= S_{top} + S_{bottom} + S_{lateral} = 2\pi r^2 + 2\pi r^2 + 2\pi rl = 2\pi\times 1.8^2 + 2\pi\times 1.8^2 + 2\pi\times 1.8\times 100 = 1171.688$ nm$^2$

Considering that both GNSs and GNWs are formed by Au atoms, we have:

The volume of GNS: $V_{GNSs} = 4\pi r^3/3 = 1436.755$ nm$^3$

The volume of GNWs: $V_{GNWs} = 4\pi r^3/3 + \pi r^2 h = 4\times\pi\times 1.8^3/3 + \pi\times 1.8^2\times 100 = 1042.305$ nm$^2$

$S_{GNSs} / V_{GNSs} = 0.429$

$S_{GNWs} / V_{GNWs} = 1.124$

For GNWs longer than 100 nm, the $S_{GNWs} / V_{GNWs}$ will be even greater. For GNSs and GNWs on the same mass basis, we can conclude that GNWs have a higher surface area than GNSs do.

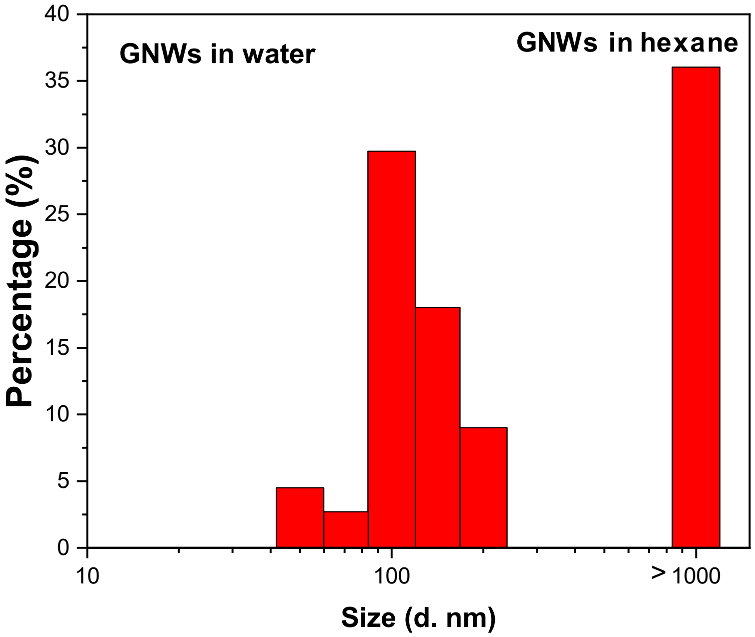


**Figure S1.** Length distribution of as-synthesized GNWs in hexane and phospholipid-coated GNWs in water. Unmodified GNWs are all greater than 1 μM in length. After phospholipid coating, the length of the GNWs are reduced to ~100 nm.

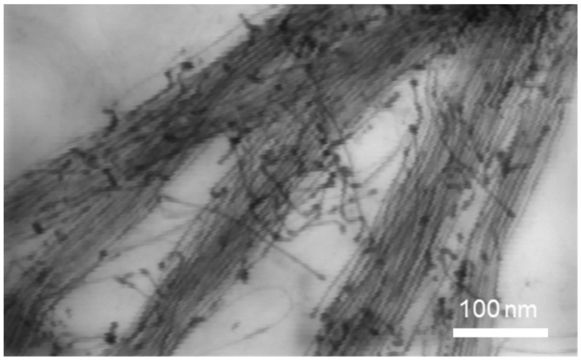


**Figure S2**. TEM image of phospholipid-coated GNWs in water.

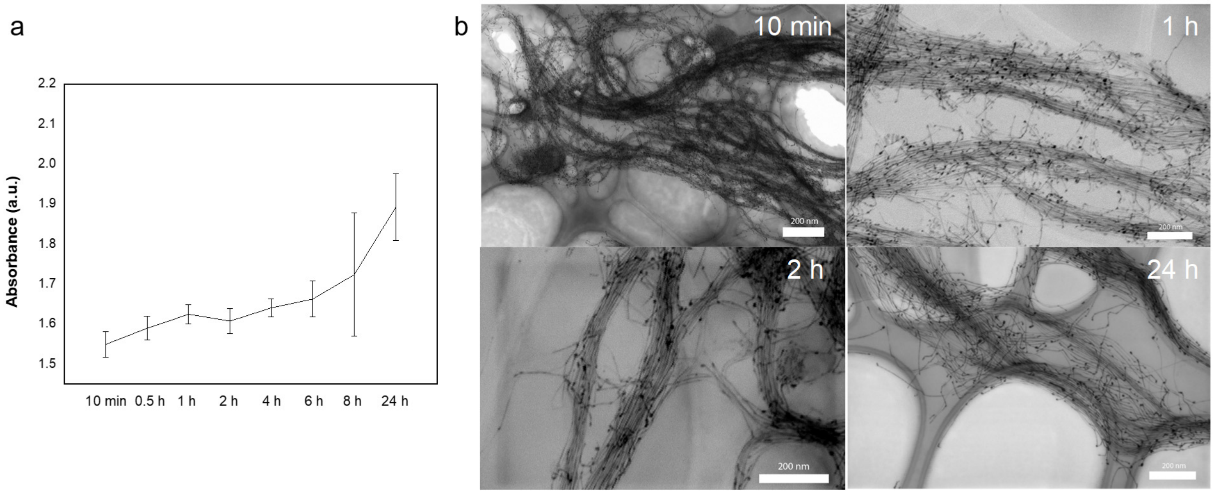


**Figure S3.** Degradation of GNWs in the presence of glutathione (1 mM). a) Gradual increase of solution absorbance at 530 nm, which is attributed to the degradation of GNWs and the generation of shorter nanowires and nanoparticles. b) TEM images showing gradual degradation of GNWs, which produced shorter nanowires along with spherical nanoparticles.
